# Head skeleton malformations in zebrafish (*Danio rerio*) to assess adverse effects of mixtures of compounds

**DOI:** 10.1007/s00204-018-2320-y

**Published:** 2018-10-04

**Authors:** Yvonne C. M. Staal, Jeroen Meijer, Remco J. C. van der Kris, Annamaria C. de Bruijn, Anke Y. Boersma, Eric R. Gremmer, Edwin P. Zwart, Piet K. Beekhof, Wout Slob, Leo T. M. van der Ven

**Affiliations:** RIVM: National Institute for Public Health and the Environment, Antonie van Leeuwenhoeklaan 9, PO Box 1, 3721 MA Bilthoven, The Netherlands

**Keywords:** Mixture toxicology, Cumulative assessment groups, Head skeleton, Craniofacial malformations, Zebrafish embryos

## Abstract

The EU-EuroMix project adopted the strategy of the European Food Safety Authority (EFSA) for cumulative risk assessment, which limits the number of chemicals to consider in a mixture to those that induce a specific toxicological phenotype. These so-called cumulative assessment groups (CAGs) are refined at several levels, including the target organ and specific phenotype. Here, we explore the zebrafish embryo as a test model for quantitative evaluation in one such CAG, skeletal malformations, through exposure to test compounds 0–120 hpf and alcian blue cartilage staining at 120 hpf, focusing on the head skeleton. Reference compounds cyproconazole, flusilazole, metam, and thiram induced distinctive phenotypes in the head skeleton between the triazoles and dithiocarbamates. Of many evaluated parameters, the Meckel’s–palatoquadrate (M–PQ) angle was selected for further assessment, based on the best combination of a small confidence interval, an intermediate maximal effect size and a gentle slope of the dose–response curve with cyproconazole and metam. Additional test compounds included in the CAG skeletal malformations database were tested for M–PQ effects, and this set was supplemented with compounds associated with craniofacial malformations or cleft palate to accommodate otherwise organized databases. This additional set included hexaconazole, all-trans-retinoic acid, AM580, CD3254, maneb, pyrimethanil, imidacloprid, pirimiphos-methyl, 2,4-dinitrophenol, 5-fluorouracil, 17alpha-ethynylestradiol (EE2), ethanol, 2,3,7,8-tetrachlorodibenzo-*p*-dioxin (TCDD), PCB 126, methylmercury, boric acid, and MEHP. Most of these compounds produced a dose–response for M–PQ effects. Application of the assay in mixture testing was provided by combined exposure to cyproconazole and TCDD through the isobole method, supporting that in this case the combined effect can be modeled through concentration addition.

## Introduction

Risk assessment of mixtures of compounds (cumulative risk assessment, CRA) is a much debated and highly relevant issue, because humans (and environmental organisms) are always exposed to combinations of (classes of) compounds. No harmonized strategy exists to predict the effects of compounds in the context of a mixture (Kienzler et al. 2016), and various paradigms for combined effects have been proposed, which, when related to dose, include simple dose addition, and infra- and supra-additivity models (Cassee et al. 1998). Supra-additivity, including synergism, as a particular deviation from dose addition, is of most concern in the regulatory context, but is only rarely observed at relevant exposure levels in ecological (Cedergreen 2014) and even less so in human (Hernandez et al. 2017) hazard assessment. The concentration or dose addition model (Bosgra et al. 2009) and simple similar action model are the most commonly known approaches for mixture risk assessment (EFSA 2013a), in which one compound is expressed in concentration equivalents of another compound, based on their relative toxicological potency.

For a practical approach in risk assessment of mixtures, the European Food Safety Authority (EFSA) proposed applying dose addition as default, and further, to limit the number of chemicals that need to be considered in actual mixtures, to group chemicals in so-called cumulative assessment groups (CAGs) (EFSA 2013a). These groups are based on their toxicological properties, including target organ/system (level 1), specific toxicological effect (phenotype; level 2), and mode/mechanism of action (levels 3–4). This allows the exclusion in CRA of large numbers of chemicals that do not have the toxicological end point of interest in common. This approach is further explored in the EU-funded EuroMix project, with focus on the CAG developmental toxicity, and skeletal malformations as specific level 2 toxicological effect (Nielsen et al. 2012). When focusing on the head skeleton, common causes, e.g., effects on the fate of neural crest cells, which are a major contributing cell population, may lead to a variety of craniofacial anomalies (Menegola et al. 2006). Such anomalies are commonly observed in humans and affect approximately 1 in every 1000 individuals (CDC 2017). In the experimental setting, such anomalies, including cleft palate and facial dysmorphia, can be induced with compounds representing various chemical classes, such as the triazole and dithiocarbamate (DTC) fungicides (Nielsen et al. 2012; van Boxtel et al. 2010; Wolterink et al. 2016). Such compound effects are not always particularly specific, and therefore compound databases for skeletal malformations and cleft palate show major overlaps (Kyriakopoulou et al. 2016; Nielsen et al. 2012).

The zebrafish (*Danio rerio*) is increasingly used as a model in human toxicological research and is highly suitable to assess the effects of compounds on bone and cartilage structures. The early stages of development, involving skeletal formation, are highly conserved between species and, therefore, the zebrafish model is considered relevant to assess the effects on skeletal malformations in humans. Specific advantages of the model are that zebrafish are transparent during the early stages of development, and the embryos are exempted from registration under European animal experimentation legislation up to 5 days post-fertilization. More generally, the OECD236 test guideline is used to assess (sub)acute toxicity to zebrafish and, for our purpose, we expanded this guideline with a more detailed toxicological scoring system (Hermsen et al. 2011). In this way, the zebrafish embryo toxicity assay (ZFET) is a useful tool for systematic analysis of toxic concentration ranges and to detect whether compounds can affect head structures (Hermsen et al. 2011). This protocol served as a basis for a zebrafish test to assess skeletal malformations during development. Chondrogenesis is already present at embryonic day 2 in zebrafish embryos, and supportive cartilage structures are present at the time of hatching (3dpf) (Kimmel et al. 1998). The head skeleton can be readily visualized in 120 hpf embryos, particularly after application of alcian blue cartilage staining (van Boxtel et al. 2010). The aim of the research described in this paper was to develop and optimize a quantitative assessment protocol for malformations in the head skeleton of the zebrafish embryo, in view of applicability for assessment of combined effects of chemicals in EFSA CAG skeletal malformations. This was done through analysis of the effects of two different compound classes of pesticides with known effects on skeletal development, which are relevant in view of human exposure. Triazole fungicides affect development through inhibition of CYP26 and subsequent disruption of the retinoic acid balance; this may lead to altered specification, migration, differentiation and/or maturation of neural crest cells, which contribute to formation of cartilage and bone in the head skeleton and thus to malformations in these tissues (Hermsen et al. 2012; Menegola et al. 2006). Such effects of triazoles are well characterized (e.g., Di Renzo et al. 2011; Machera 1995; Menegola et al. 2006), and representative compounds of this chemical class were therefore selected as reference compounds for this study. Dithiocarbamate pesticides (DTCs) also affect bone and cartilage formation in zebrafish, through other mechanisms, i.e., by binding to copper and thus inhibiting cupro-enzymes, by inhibiting lysyl oxidase activity resulting in loss of connective tissue, and possibly also by altering intracellular functions (Grau-Bové et al. 2015; van Boxtel et al. 2011). DTCs were, therefore, selected as the second reference group. Assessment of head malformations was further tested with a range of other compounds which have been associated with malformations in the head skeleton (Nielsen et al. 2012, report and accompanying CAPEG database at https://efsa.onlinelibrary.wiley.com/doi/10.2903/sp.efsa.2012.EN-269). Finally, a mixture was tested to assess the suitability of the protocol for this application.

## Materials and methods

*Danio rerio* (zebrafish), originally obtained from commercial wild-type import (Ruinemans Aquarium BV, Monfoort, The Netherlands), and maintained and propagated in our facility for more than ten generations, were kept in 7.5 L ZebTec tanks (Tecniplast S.p.A, Buguggiate, Italy). The temperature was maintained at 27.5 ± 1 °C, the pH at 7.5 ± 0.5 and the conductivity at 500 ± 100 µS. The photoperiod was 14 h light and 10 h dark and light intensity was gradually dimmed or increased over a 30-min period. The fish were fed twice a day with SDS 100, 200, 400 or small granules (Special Diet Services, Essex, UK) depending on the age of the fish, and supplemented with defrosted *Artemia salina (*artemia; Ruto Frozen FishFood Zevenhuizen, The Netherlands; once daily for adults) or live artemia (three times per day for larvae and young juveniles; in-house culture from artemia eggs).

Four days prior to spawning, females were separated from males and fed a high protein diet (artemia three times/day). The afternoon before spawning, two females and two males were introduced into breeding tanks. Immediately after spawning, which was initiated by morning light, fertilized eggs were collected with a sieve and rinsed thoroughly with Dutch Standard Water (DSW) (Hermsen et al. 2011). Eggs were transferred to separate Petri dishes per breeding unit, and the quality was checked under a microscope. Batches with less than 20% coagulated eggs and limited egg deformations were pooled and used for experiments. Eggs between 4- and 64-cell stage were exposed in 24-well plates (1 egg per well) containing 2 mL test medium. The test medium consisted of DSW with 0.1% DMSO (Merck, Darmstadt, Germany) and, except for the zero baseline condition, a test compound with a concentration in the range given in Table 1. Ten replicate embryos were used per condition (solvent control or test concentration). All test compounds were obtained from Sigma-Aldrich, Zwijndrecht, Netherlands, except for CD3254 (Santa Cruz Biotechnology, Huissen, The Netherlands), ethanol (Merck), and PCB 126 (Chiron AS, Trondheim, Norway). For dose–response modeling, four to five half-logarithmic dilutions were derived from the highest concentration, and one blank control included, producing a total of six to seven conditions per experiment (exceptionally less, down to 4; see “Results”). The highest concentration was chosen at a level slightly below the lethal toxic effects, as determined in a preceeding OECD-236 guideline-based range-finding zebrafish toxicity test (ZFET; Hermsen et al. 2011 and explained below). In some cases, the concentration series were adjusted to optimize the dose–response curve. Exposure was static, i.e., without medium refreshment during the 5-day exposure period. This assumption was supported by the observed toxicity in the dose-range finding tests.

**Table 1:** Used compounds and test concentrations

Compound	Code	Chemical class	CAS	Test concentration range (µM)^a^
Cyproconazole	cyp	Triazole	94361-06-5	3–300
Flusilazole	flu	Triazole	85509-19-9	1–60
Hexaconazole	hex	Triazole	79983-71-4	1–60
All-trans retinoic acid	RA	Retinoid	302-79-4	0.01–1
CD3254	CD	Retinoid	196961-43-0	0.1–3
AM580	AM	Retinoid	102121-60-8	0.001–0.03
Metam	met	Dithiocarbamate	137-42-8	0.1–10
Thiram	thir	Dithiocarbamate	137-26-8	0.003–1
Maneb	man	Dithiocarbamate	12427-38-2	0.1–10
Pyrimethanil	pyr	Anilinopyrimidine	53112-28-0	0.3–30
Imidacloprid	imi	Neonicotinoid	138261-41-3	0.3–1000
Pirimiphos-methyl	pir	Organophosphate	29232-93-7	0.3–10
2,4-Dinitrophenol	2,4-DNP	Dinitrophenol	51-28-5	3–30
5-Fluorouracil	5FU	Pyrimidine analog	51-21-8	0.1–100
17Alpha-ethynylestradiol	EE2	Synthetic steroid hormone	57-63-6	0.3–10
Ethanol	EtOH	Alcohol	64-17-5	0.1–30
2,3,7,8-tetrachlorodibenzo-p-dioxin	TCDD	Dioxin	1746-01-6	0.0001–0.01
PCB 126	PCB126	Polychlorinated biphenyl	57465-28-8	0.3–10
MEHP	MEHP	Phthalate	4376-20-9	3–100
Methylmercury	MeHg	Organometallic compound	0115-9-3	0.01–1
Boric acid	ba	Boron derivative	10043-35-3	0.03–10

^a^See text for explanation of test concentration ranges

During exposure, embryos were kept in an incubator at 27.5 °C, with a 14:10 h light: dark cycle. After 3 and 5 days post-fertilization (dpf) the development and teratological effects of the embryos were evaluated under a light microscope as described previously (Hermsen et al. 2011). Development was scored using an integrative semi-quantitative scoring system (General Morphology Score, GMS) for specific developmental end points, including detachment of tail, formation of somites, development of eyes, movement, heartbeat, blood circulation, pigmentation of head–body, pigmentation of tail, pectoral fin, protruding mouth, and hatching. In addition, teratological effects were scored as present or absent as a total teratology score, considering pericardial edema, yolk sac edema, eye edema, malformation of the head, absence/malformation of sacculi / otoliths, malformation of tail, malformation of heart, modified chorda structure, scoliosis, rachischisis, and yolk deformation. Of these, malformation of the head was also analyzed separately as a key end point in this investigation.

Alcian blue staining was used for optimal visualization of cartilage structures in the head of the embryos (head malformations). The method was based on previous research (Cohen et al. 2014; Kimmel et al. 1998; van Boxtel et al. 2010), which was adapted as follows. After 5dpf, embryos were euthanized by rapid cooling of the plates on ice. The exposure medium was then removed and the embryos were fixed for 2 h in 4% paraformaldehyde (PFA) at room temperature. Subsequently, fixed embryos were washed three times with PBS for 10 min and bleached with 3% H_2_O_2_ and 0.5% KOH for 35 ± 5 min. After three wash cycles with PBS, embryos were stained overnight at 4 °C with 0.01% alcian blue in 60 mM MgCl_2_ and 70% ethanol. After staining, embryos were washed with 80% ethanol/10 mM MgCl_2_, 50% ethanol/10 mM MgCl_2_ and 25% ethanol, respectively. To reduce staining of soft tissues, embryos were bleached again with 3% H_2_O_2_ and 0.5% KOH for 15 ± 5 min. Embryos were washed with 25% glycerol and 0.1% KOH and stored in 50% glycerol in 0.1% KOH. Stained embryos were positioned in this storage solution in slits of a silica gel block or a 3D-printed device (Wittbrodt et al. 2014) and the head photographed in a ventral–dorsal and a lateral view. These photos were analyzed with Adobe Photoshop by measuring distances and angles related to the head skeleton anatomy. Malformations were quantified as described previously (Cohen et al. 2014), although with additional parameters (see “Results”), and additional visual assessment applied where applicable.

### Statistical analyses

R (version 3.2.3) with work package PROAST (version 60.8 or 65.5) was used to analyze the morphology and teratology score with the benchmark dose–response approach (Slob 2002). In this report, dose–response is used as a standard term, although it refers to concentrations. The benchmark concentrations (BMC) were calculated from the dose–response curves, with a predefined critical effect size (CES) of 5% increase. The data were fitted using a nested family of models with an increasing number of parameters. The exponential and Hill models were used for the morphology score and all quantitative measurements in the head skeleton. Four models were fitted for the teratology score, namely log-logistic, Weibull, log-probit and gamma. The log-likelihood was calculated to determine the goodness of fit. The model which used the lowest number of parameters was selected. For malformations in the cartilage structure, the distances, ratios and angles of the defined parameters were fitted using the exponential model with the following equation: $y=a\{ (c - \left( {c - 1} \right)\exp ( - b{x^d})$, in which *y* is the response and *x* the concentration. The parameters a, b, c and d are constants to be fitted by the PROAST software. Parameter *a* expresses the response of the controls (background value); the *b* parameter relates to the potency of the tested compound (sensitivity); parameter *c* reflects the maximum response; and parameter *d* indicates the steepness of the curve (Slob 2002).

The PROAST software was also used to calculate relative potency factors (RPF), which can be done through combined analysis of two sets of dose–response data of two compounds (in terms of RPF). The final mixture analysis in PROAST is based on a combined assessement of two compounds in the combinations *A*_1–2−*n*_ + 0 and 0 + *B*_1–2−*n*_, for single compounds *A* and *B*, respectively, and *A*_1–2−*n*_ + *B*_1−2−*n*_ for the mixture. The output is a single dose–response curve based on the two single compounds, and including or excluding the data points of the mixture (see paragraph “Binary mixtures”).

### Binary mixtures

**Fig. 1:**
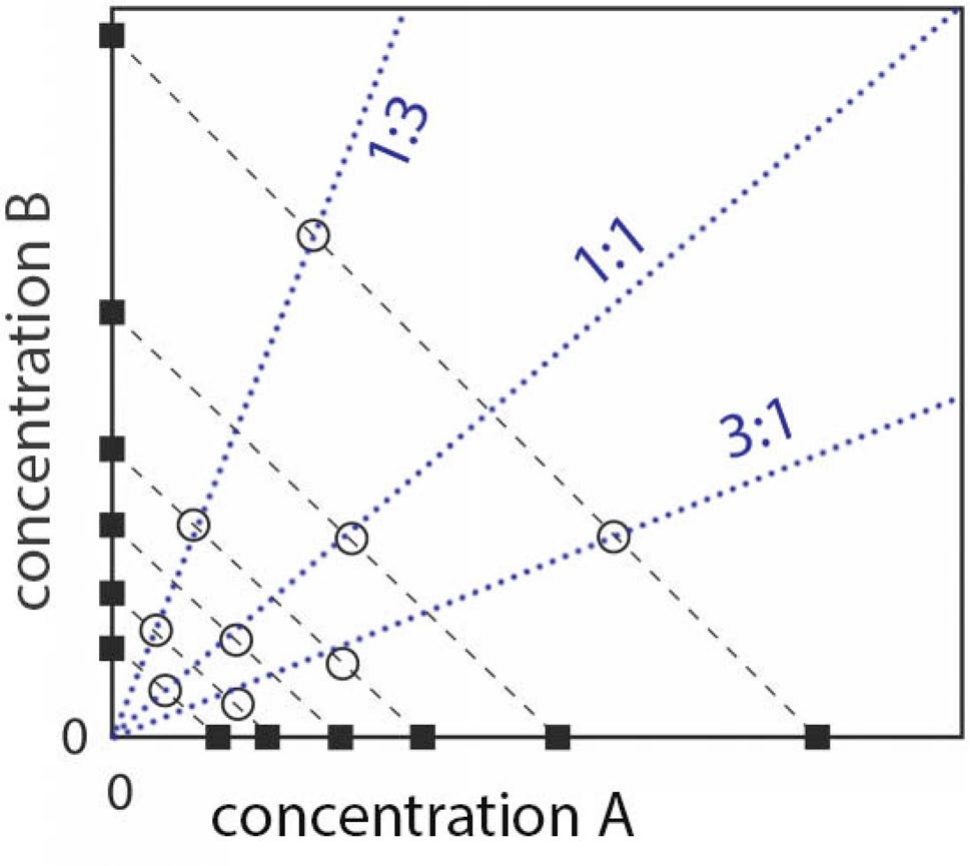
Isobole diagram [after (Kienhuis et al. 2015)]. Equipotent concentrations of compounds A and B are defined from single compound exposures (closed square symbols) and connected through isoboles (black dashed lines). The combined exposure has mixtures (open circles) of equal contributions of equipotent concentrations of each compound (1:1, middle blue dotted line), excess of compound *A* (3:1, right dotted line), and excess of compound *B* (1:3, left dotted line). (Color figure online)

Data from the individual compounds were used to select concentrations for designing a binary mixture using the isobole approach. The concentrations of the second compound *B* were expressed as equivalents of the first (index, reference) compound *A*, thereby correcting for the differences in potency using a relative potency factor (RPF) (Cassee et al. 1998; Kienhuis et al. 2015): RPF = BMC_*B*_/BMC_*A*_, in which BMC_*B*_ is the benchmark concentration of the chemical of interest and BMC_*A*_ is the benchmark concentration of the index compound. In the mixture, the two compounds are combined in 1:1 ratios of equipotency, and in excess of each compound (1:3 and 3:1 ratios), derived from isoboles (lines of equipotency in the diagram, Fig. 1), to account for dominance effects of either of the two compounds. As a rule, the various mixture concentrations were tested together with repeat concentrations of the single compounds (and recalculate the actual RPF), to account for effectivity differences between experiments, and distributed to mainly cover the intermediate part of the dose–response curve. In the analysis, the single compounds were plotted along a single dose–response curve, because the software will recalculate the concentrations of the second compound to equivalents of the reference compound using the actual RPF. If dose addition applies to the mixture, its data points will not show a systematic deviation of the curve derived from the single compounds. In cases of less or more than dose addition, the mixture data points will show a shift to either the right or the left, respectively, of the single compounds curve.

## Results

### Phenotype of head skeleton malformations induced by triazoles and dithiocarbamates

Two triazoles (cyproconazole, flusilazole) and two dithiocarbamates (metam, thiram) were used as reference for assessment of two distinctive phenotypes of head skeleton malformations. First, these compounds were tested in the ZFET assay with initiation of exposure directly after fertilization and assessment of general developmental morphology benchmarks and teratogenicity at 3 and 5dpf. The analysis revealed a concentration-related decrease of the general morphology score and an increase in teratogenicity for all four compounds. Malformation of the head, which is a contributing parameter to the teratology score, was analyzed separately as a specific target factor and also showed a concentration-dependent increase. The teratogenic effect of cyproconazole and flusilazole mainly consisted of pericardial edema and heart malformations. The most predominant teratological effects of metam and thiram were modified chorda structure and scoliosis.

**Fig. 2:**
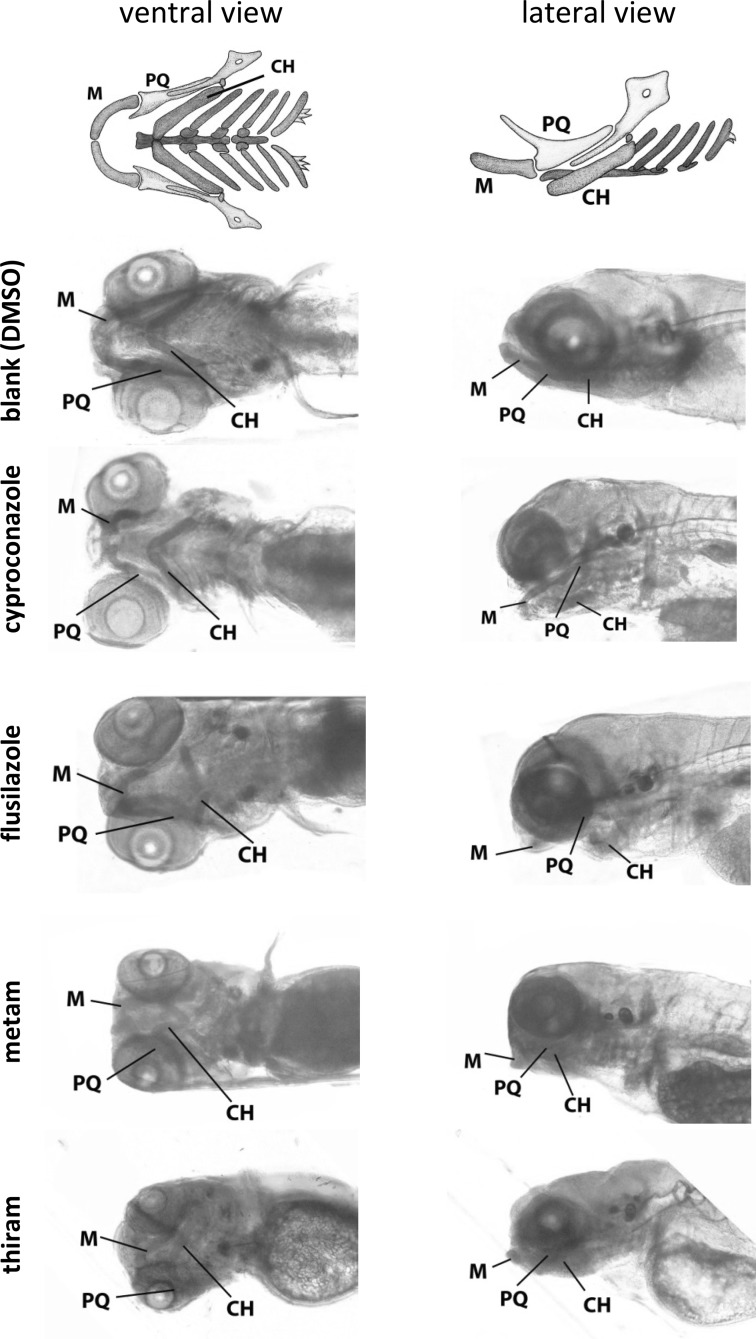
Head cartilage structures of alcian blue-stained zebrafish embryos in ventral and lateral views after exposure to DMSO only (solvent control), cyproconazole (60 µM), flusilazole (10 µM), metam (10 µM) or thiram (0.3 µM), at the highest non-lethal concentrations. The first row shows a schematic representation of the head skeleton, reproduced with permission from Kimmel et al. (1998). *M* Meckel’s cartilage, *PQ* palatoquadrate cartilage, *CH* ceratohyal cartilage (see Kimmel et al. 1998 for explanation of other structures)

Overall, the two dithiocarbamates were more potent than the two triazoles (Table 2). Within the two classes, flusilazole was more potent than cyproconazole for all three parameters, and thiram more potent than metam. The teratology score was affected in all cases, whereas GMS was not affected with metam. Malformation of the head appeared to be an important determinant of developmental, particularly teratological effects, and justified a more quantified analysis of effects in the head skeleton through enhanced visualization by staining of the cartilage. This confirmed that all four compounds induced obvious effects on head cartilage structures (Fig. 2).

**Table 2:** Benchmark concentrations of the general morphology score, teratology score and the head malformations score (component of teratology score) at 3 and 5dpf

	Three days post-fertilization		
	BMC_GMS_ (µM)	BMC_T_ (µM)	BMC_malhead_ (µM)
Cyproconazole	57.4 (50.5–64.5)	26.9 (7.8–60.1)	38.5 (18.1–62.0)
Flusilazole	6.9 (5.5–9.4)	5.6 (3.1–9.6)	16.3 (8.0–25.5)
Metam	> 10	0.9 (0.7–1.3)	> 10
Thiram	0.1 (0.05–0.2)	0.02 (0.01–0.03)	0.02 (0.04–0.05)

	Five days post-fertilization		
	BMC_GMS_ (µM)	BMC_T_ (µM)	BMC_malhead_ (µM)
Cyproconazole	58.4 (46.9–65.3)	27.5 (20.0–27.5)	53.7 (49.3–58.7)
Flusilazole	9.8 (8.9–10.7)	2.6 (1.3–6.0)	2.7 (1.3–4.3)
Metam	> 10	1.5 (0.8–2.9)	0.8 (0.07–2.5)
Thiram	0.1 (0.06–0.25)	0.02 (0.01–0.03)	0.03 (0.01–0.06)

BMC_GMS_, BMC_T_ and BMC_malhead_, benchmark concentration of the general morphology score, teratology score, and of head malformation score, respectively. From the plotted data, BMCs were (arbitrarily) derived at a 5% effect level, together with their 95% confidence interval (BMDL–BMDU)

More specifically, exposure to cyproconazole or flusilazole resulted in a shortening of the Meckel’s and the palatoquadrate cartilage and reduction of the ceratohyal angle, all in ventral–dorsal view. In the lateral view, both the Meckel’s and the ceratohyal cartilage were curved to the ventral side. Exposure to metam or thiram caused a distinct phenotype in the ventral view, characterized by a wavy pattern of all cartilage structures. In the lateral view, both the Meckel’s and the ceratohyal cartilage were curved to the ventral side similar to the triazoles.

### Parameter assessment

**Fig. 3:**
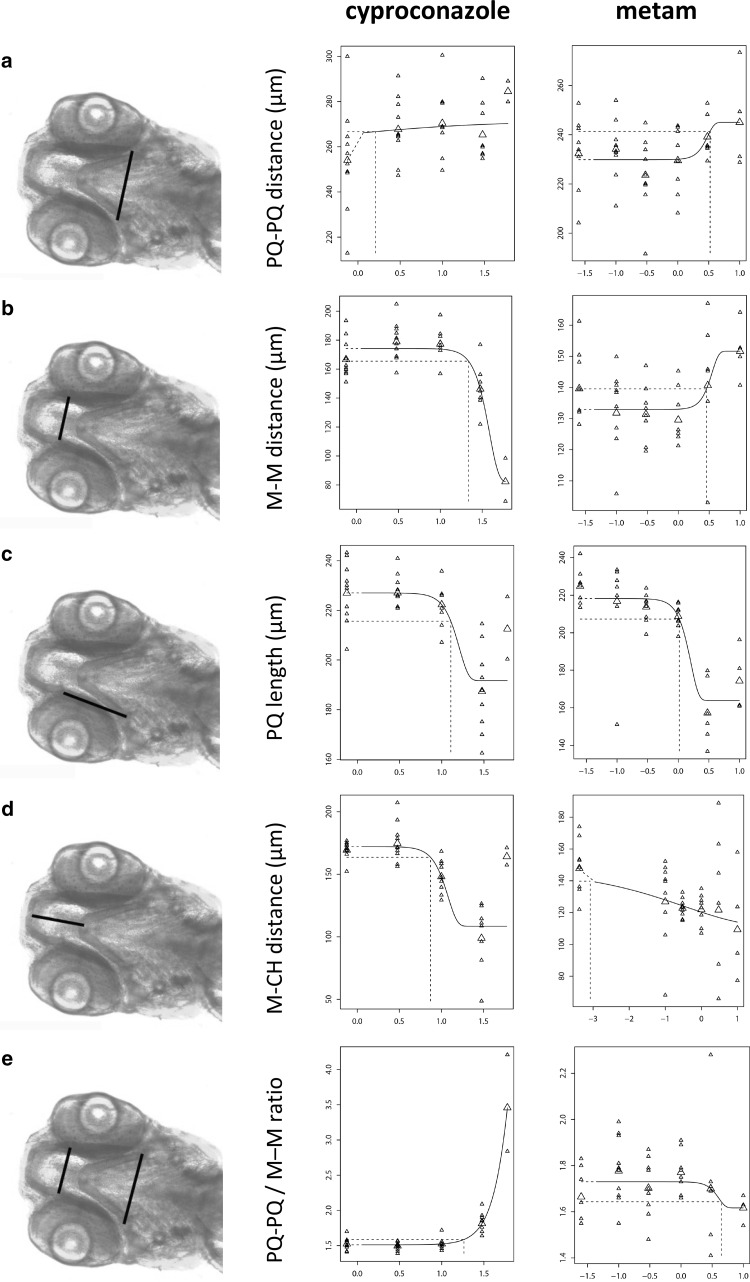

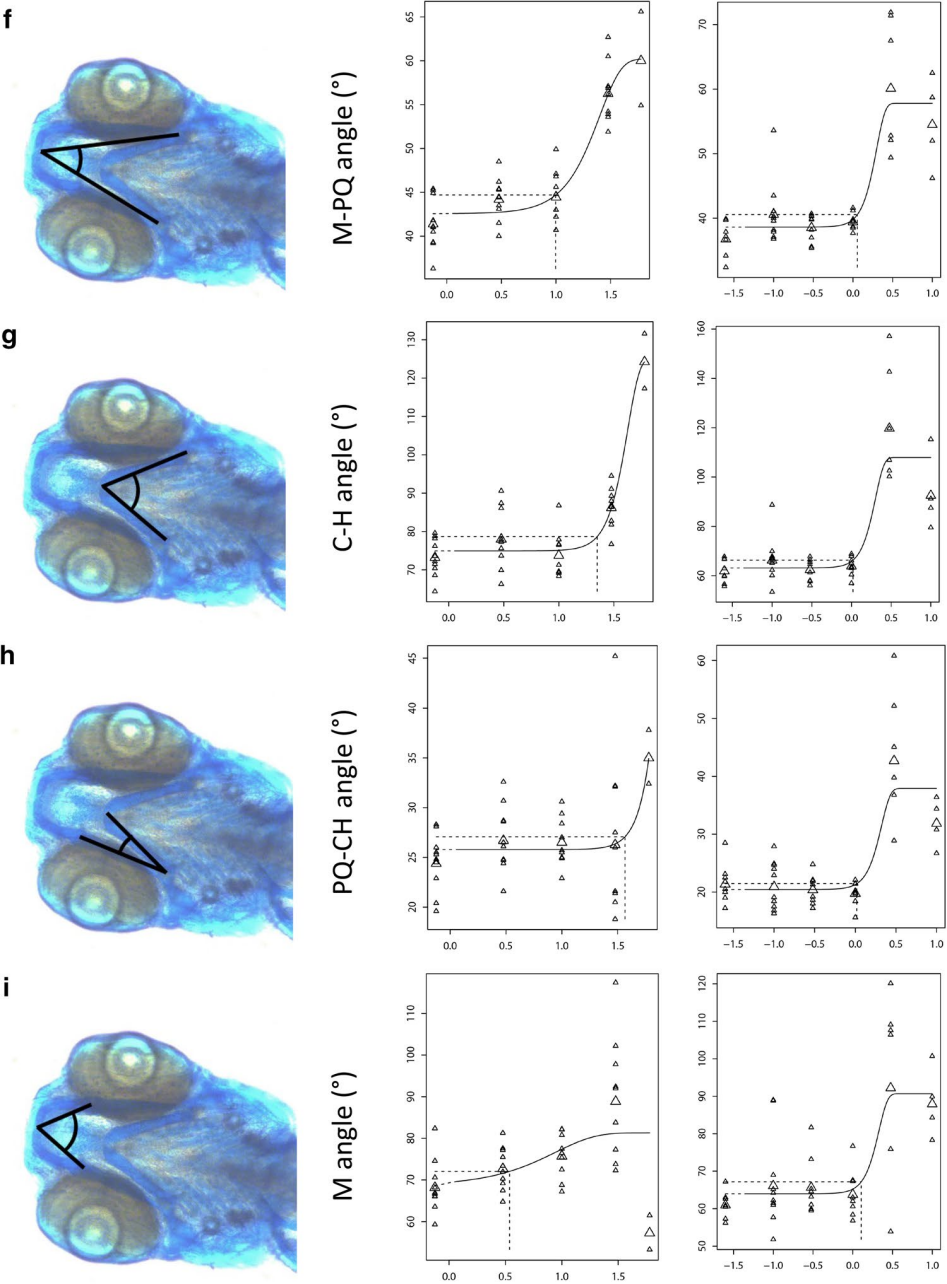
Concentration–response curves for cyproconazole and metam on several quantitative parameters. The *Y*-axis shows the response (in µm or degrees) and the *X*-axis the concentration (log10 µM). All graphs are exponentially modeled: *y* = *a* × [*c* − (*c* − 1)exp(− *bx*^*d*)], with *a*, background, *b*, sensitivity, *c*, maximal effect, *d*, slope. Small symbols, individual observations; triangles, mean values per concentration group; dotted line, BMC at 5%. **a** PQ–PQ distance; **b** M–M distance; **c** PQ length; **d** M–CH distance; **e** PQ–PQ/M–M ratio; **f** M–CH angle; **g** CH angle; **h** PQ–CH angle; **i** M angle

Cyproconazole and metam were then used to determine the most informative common parameter in the alcian blue-stained embryos for these classes of compounds, to further assess head skeleton malformations, as these two compounds produced obvious but different phenotypic effects. Both compounds induced only a slight increase (6–7%) in the distance between left and right PQ structures (Fig. 3a; Table 3). Cyproconazole induced a decrease in the distance between the M structures, whereas metam showed a (slight) increase in that distance (Fig. 3b). The length of the PQ structures and the distance from the M to the CH structure decreased for both cyproconazole and metam (Fig. 3c, d). The ratio between the distance of the PQ structures and the M structures increased after exposure to cyproconazole, but decreased after exposure to metam, as a consequence of the opposite effects for M–M distance (Fig. 3e). The measured angles were affected similarly after exposure to cyproconazole and metam, that is, increases in the M–PQ angle (Fig. 3f), the CH angle (Fig. 3g), the PQ-CH (Fig. 3h) and the M angle (Fig. 3i). However, although statistically significant, not all fits were convincing, particularly when fully determined by a deviating control (as in Fig. 3a, cyproconazole), or by the highest concentration only (as in Fig. 3h, cyproconazole), or when the fit was not determined by a consistent trend (as in Fig. 3d, cyproconazole).

**Table 3:** Dose–response parameters of cyproconazole and metam for all measured parameters

	Cypronazole				Metam			
	BMC, µM	BMC–CI, µM	Max effect, %	Slope	BMC, µM	BMC–CI, µM	Max effect, %	Slope
Distance PQ–PQ	1.32	(0–∞)	+ 6	0.25	6.7	(1.7–97.1)	+ 7	4
Distance M–M	26.4	(19.7–27.5)	− 53	4	3.0	(1.1–10.2)	+ 33	4
Length PQ	11.1	(8.9–24.9)	− 26	4	1.0	(0.7–2.3)	− 25	3.4
Distance M–CH	8.9	(0.3–11.5)	− 37	4	0.00009	(0–0.78)	− 26	0.25
Ratio PQ–PQ/M–M	17.9	(14.8–26.5)	+ 133	2.8	8.0	(2.3–∞)	− 5	4
Angle M–PQ	9.9	(4.7–25.9)	+ 41	2.2	1.1	(0.8–2.2)	+ 49	4
Angle CH	26.7	(19.4–28.1)	+ 66	4	1.4	(0.8–2.1)	+ 70	4
Angle PQ–CH	37.9	(0.3–55.1)	+ 36	4	1.8	(0.9–2.1)	+ 84	4
Angle M	3.4	(0.1–19.0)	+ 18	1.2	1.9	(0.7–2.4)	+ 41	4

Benchmark concentration (BMC) and BMC confidence interval (CI) are modeled at the 5% effect level

Dose–response characteristics for each parameter are provided in Table 3. The angles generally performed better than distances and lengths, in view of combined narrower confidence intervals, higher maximal effects, and consistency of the effect for both compounds (same direction). Among the angles, the M–PQ and CH angles showed the highest precision (narrow confidence interval) and, although the variation (confidence interval) of the CH angle was slightly smaller than of the M–PQ angle, the latter was selected for further application in view of a wider effect-determining concentration range with cyproconazole, and in view of a smaller background angle and smaller relative maximal effect, which should enable assessment of stronger responses than with the two tested compounds, within the absolute limit for both angles of 180 degrees.

### Application of M–PQ

**Fig. 4:**
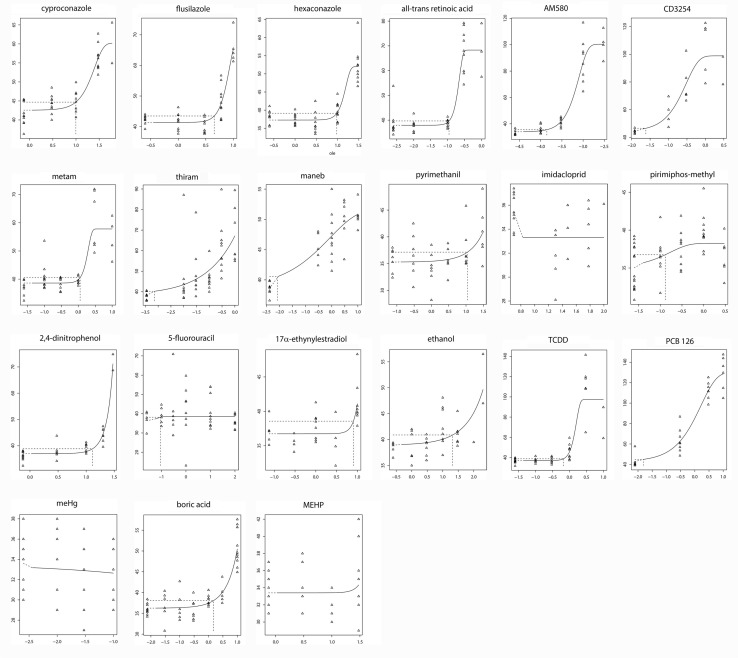
M–PQ angle dose–responses modeled by E5 − BMC: *y* = *a* × [*c*−(*c* − 1)exp(− *bx*^*d*)] (Slob 2002). All compounds were tested up to the highest sublethal concentration. X-axis, log10 compound concentrations in µM (except TCDD: nM); *Y*-axis, M–PQ angle (°); linear; small symbols, individual observations; large symbols, mean per concentration group; horizontal dotted line, 5% effect level (CES); vertical dotted line, BMC at 5% CES. BMCs, BMC confidence intervals, maximum effect sizes (c-parameter), and slopes (d-parameter) are given in Table 4

An extended set of pesticides and environmental contaminants of concern (Table 1), categorized as inducers of skeletal malformations and cleft palate (Kyriakopoulou et al. 2016; Martin and Judson 2010; Nielsen et al. 2012), were assayed for effects in the head skeleton of the zebrafish embryo. Dose–responses and some dose–response characteristics for the M–PQ angle are shown in Fig. 4 and Table 4. In this test series, 16 out of the 21 tested compounds induced an effect higher than 10% (see Max effect size, Table 4), i.e., all conazoles, retinoids, dithiocarbamates, pyrimethanil, 2,4-DNP, EE2, ethanol, TCDD, PCB 126, and boric acid, whereas the other six compounds induced no or only a limited effect, which was confirmed by overall visual assessment of the embryos (not shown). In the case of hexaconazole, EE2, and ethanol, a statistically significant dose–response depended on an effect in the highest test concentration only, which was always near the overall toxicity concentration. Most of the effective compounds had a narrow confidence interval (less than a factor 10 difference between the upper and lower bound). Within the group of effective compounds, the overall most potent chemical classes were the dioxin and retinoid receptor agonists, and the dithiocarbamates, then triazoles, boric acid and EE2, and finaly 2,4-DNP and ethanol.

**Table 4:** Dose–response parameters of the test compounds

	BMCs @5% (µM)	Confidence interval	Max effect size (%)	Slope
Cyproconazole	9.9	4.7–17.6	+ 41	2.2
Flusilazole	4.5	3.7–5.0	+ 68	4.0
Hexaconazole	9.6	6.6–14.4	+ 40	4.0
All-*trans* retinoic acid	0.11	0.08–0.13	+ 80	4.0
AM580	0.00014	0.000092–0.00021	+ 196	2.1
CD3254	0.024	0.0055–0.068	+ 123	1.3
Metam	1.1	0.8–1.44	+ 49	4.0
Thiram	0.00071	0.00003–0.013	+ 76	0.37
Maneb	0.0084	0.00018–0.092	+ 34	0.4
Pyrimethanil	10.5	1.5–26	+ 14	1.0
Imidacloprid	0.000001	0–inf	− 6	0.3
Pirimiphos-methyl	0.13	0–0.61	+ 9	1.0
2,4-Dinitrophenol	13.0	10.7–15.3	+ 95	3.1
5-Fluorouracil	0.092	0–inf	+ 6	2.03
EE2	8.1	2.9–9.2	+ 12	4
Ethanol	20.6	1.2–172	+ 29	0.7
TCDD	0.00066	0.00045–0.0008	+ 164	3.9
PCB 126	0.016	0.0029–0.047	+ 200	0.79
Methyl mercury	− 1.0		− 1.8	0.25
Boric acid	1.5	0.6–3.0	+ 40	1.0
MEHP	34.7		0	4.0

Confidence intervals are graphically represented in Fig. 6a

**Fig. 5:**
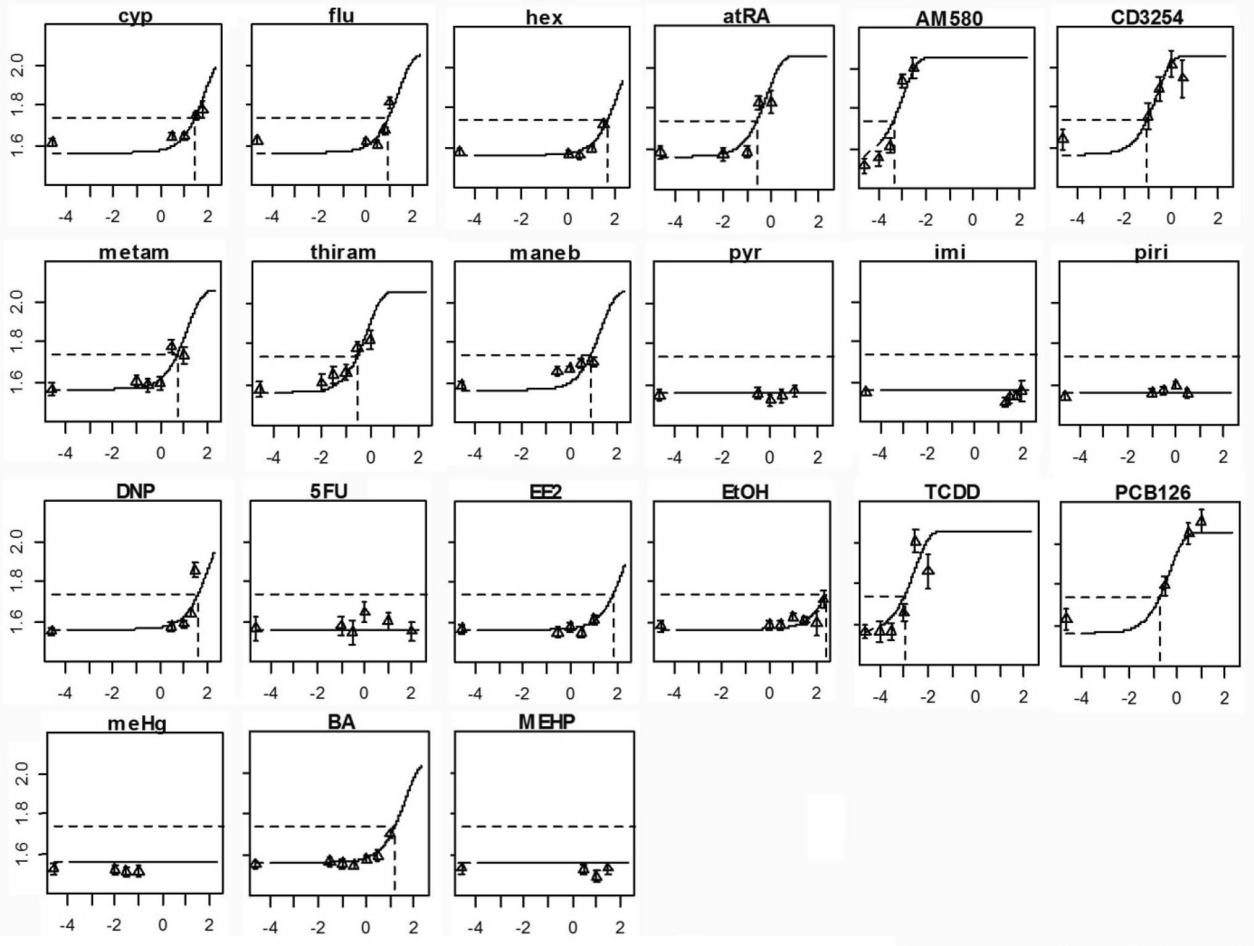
Dose–response analysis of the same set of compounds of Fig. 4, but here with the combined data as a single input for analysis. All single compound curves have the same shape, enabling precise calculation of relative potencies. Potencies and BMC variation are shown in Fig. 6b. All concentrations are in log10 µM (*X*-axis), and the response is in log10 M–PQ degrees (*Y*-axis)

**Fig. 6:**
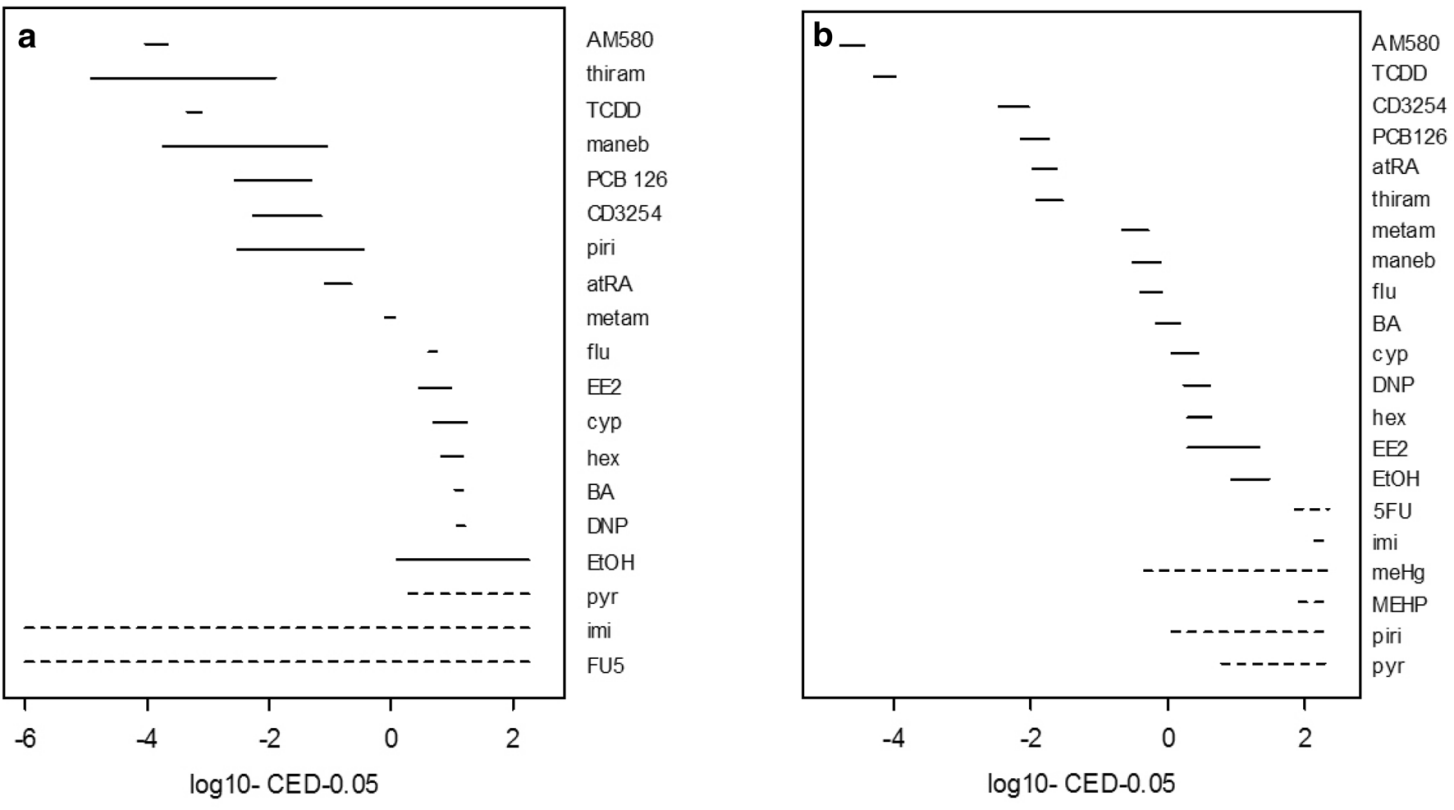
BMC confidence intervals (solid lines) along the exposure concentration (X-axis, log10 µM), derived from the analysis in Figs. 4a and 5b. The dotted lines indicate that there is no bound on either one or both sides (zero or infinite value). Confidence intervals in **b** are smaller than in **a**. The retinoic acid and dioxin receptor agonists cluster as most potent compounds in **b**, and together with the dithiocarbamates in **a** (at the left side in both graphs)

This analysis suggested that various chemical classes had distinctive dose–response characteristics, for instance through separation of retinoic acid and dioxin receptor agonists by a distinctive high maximum effect. However, for the purpose of relative potency determination among individual compounds, which is a major purpose of single compound dose–response analysis in the context of combined effects, the description of a generally applicable dose–response fit is preferred. To this aim, the combined data set was analyzed in a single run, thus generating identically shaped dose–response curves for all compounds (Fig. 5). The input of such a large dataset also leads to smaller confidence intervals per compound, compared to the dose–response analysis per compound (Fig. 6). The generation of parallel dose–response curves enables a precize calculation of relative potency factors, as illustrated in the case of cyproconazole and TCDD in the next section.

### Application of M–PQ analysis in a combined exposure experiment

**Fig. 7:**
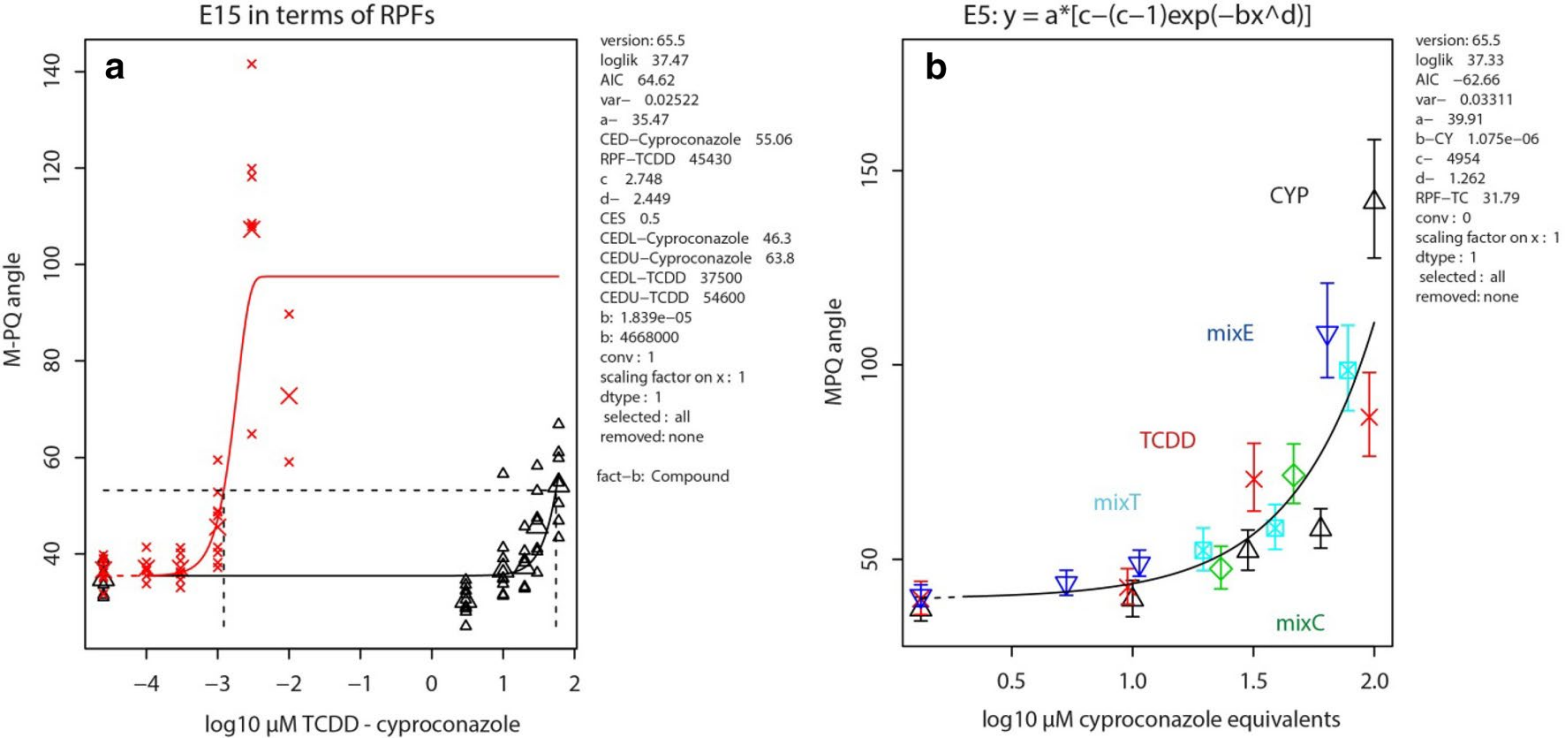
**a** Analysis of RPF 45,430 of TCDD (red crosses and fit) relative to cyproconazole (black triangles and fit); small and large symbols, individual observations and means per concentration group, respectively. **b** Mixture analysis of the two compounds. The new RPF reads 31,792, accounting for interexperimental variation between the original single compound, results in A and the single compound concentrations used in the mixture experiment (TCDD, red crosses; cyproconazole, black upward triangles). None of the three separately displayed mixture conditions (equipotency mixE [dark blue downward triangles], relative excess cyproconazole mixC [green diamonds], and relative excess TCDD mixT [light blue squares]) indicate deviation from the overall dose–response fit. All conditions in **b** given as mean ± standard deviation. (Color figure online)

Based on their single compound dose–response curves, and representing different chemical classes, cyproconazole and TCDD were selected to assess their combined effect on head skeletal malformation in the zebrafish embryo. As a first step, the RPF of TCDD compared to the index compound cyproconazole was analyzed using the results of dose–response analyses of the two single compounds in a single run (Fig. 7a), which finds the optimal, identically shaped fit with parallel slopes for the two compounds (similar to the approach in Fig. 5). The thus generated RPF estimate of 45,430 (CI 37,500–54,600) was then used to design a mixture experiment, along the lines described in Methods (Fig. 1, for this experiment further developed in Table 5). In the combined experiment (Fig. 7b), exposure to single compounds is repeated along with the mixture conditions, to account for interexperimental variation. In this case, this produces a new RPF estimate (31,792; CI 26,900–37,800) deviating from the initial RPF, which is indicative of a study replication error (e.g., due to relative pipetting imprecision). The effect curves of the single compounds overlap, as TCDD is expressed in cyproconazole equivalents following conversion through the actual RPF. The analysis further shows that the mixture conditions do not consistently deviate from the overall dose–response curve (Fig. 7b), leading to the conclusion that the two compounds in the combined exposure act in concentration addition. None of the excess ratios suggested that one of the two compounds dominated the effect. The highest concentration of the single compounds and the two highest mixture concentrations (Table 5) appeared to be lethal and could therefore not be included in the analysis.

**Table 5:** Mixture design cyproconazole + TCDD

Ratio	Final concentration (µM CYP equivalents)	CYP concentration (µM)	TCDD concentration (nM)
		0	0
1:0		10–30–60–100–300	0
0:1	14–45–136–454	0	0.3–1–3–10
1:1	6.25	3.125	0.069
1:1	12.5*	6.25	0.139
1:3	25	6.25	0.416
3:1	25	18.75	0.139
1:3	50	12.5	0.832
3:1	50	37.5	0.277
1:1	75	37.5	0.832
1:3	100	25	1.665
3:1	100	75	0.555
1:1	200	100	2.220

TCDD concentrations can be expressed as µM_cyproconazole (CYP) equivalents through conversion with the RPF (nM_TCDD/1000*45,430), or target concentrations of TCDD can be calculated the other way round. As an example (*), the final concentration of 12.5 µM cyproconazole equivalents in a 1:1 mixture is composed of 12.5/2 = 6.25 µM cyproconazole and 12.5/2/45430 (RPF) = 0.139 nM TCDD. The range of mixtures is designed to cover the intermediate part of the single compound dose–response curves

## Discussion

The European Food Safety Authority (EFSA) proposed a strategy for cumulative risk assessment (CRA), which limits the number of compounds to consider in a mixture to only those that are relevant for a specific end point, i.e., compounds assigned to specific CAGs (EFSA 2013b). This strategy has been adopted in the EuroMix project, and in this paper we elaborated on a method for quantitative assessment in one such CAG, skeletal malformations. As a model, we focused on malformations in the head skeleton in the zebrafish embryo, which enables effective visualization of the targeted end point. Therefore, a standardized ZFET assay (Hermsen et al. 2011) was extended to 5 days and alcian blue staining of the cartilage applied to allow quantification of abnormalities.

In the ZFET assay, the dithiocarbamates metam and thiram were more potent than the triazole fungicides cyproconazole and flusilazole regarding teratogenicity scores, and overall, thiram was the most potent compound regarding GMS. The observed severe teratogenic effects induced by the dithiocarbamates, particularly wavy patterns of all head cartilage structures and in the chorda, confirm other observations in fish (Tilton et al. 2006; van Leeuwen et al. 1985), and are in line with observed chondroskeletal malformations in chicken (Orth and Cook 1994). On the other hand, the effect of the triazoles was mainly characterized by shortening of the Meckel’s structure. This difference in phenotype may be attributed to the different modes of action of these two classes of compounds, their interference at a different developmental window, their different toxicokinetic properties, etc. (Menegola et al. 2006; van Boxtel et al. 2010). An example of the relevance of the sensitive developmental window, which may be associated with different targeted processes, is found in the induction of cleft palate in C57BL/6N mice, which shows different sensitive windows with retinoic acid (gd 10 more sensitive) compared to TCDD (gd 12 more sensitive) (Birnbaum et al. 1989). Different classes of compounds thus apparently target different developmental processes along the closure pathway of the palate. This observation reveals two factors to consider when combining compounds of different chemical classes in a CAG. Firstly, using a common denominator of effect may mask sufficient refinement at CAG level 2 (combining fruits instead of separating apples and oranges). Secondly, specific factors in the applied model, such as its complexicity, covered time frame of development, or species of origin, may determine the inclusion of different classes of compounds in a CAG. These and other considerations for optimization of mixture testing and risk assessment strategies, including CAG refinement at the level of mode and mechanism of action, have been discussed extensively elsewhere (EFSA 2013a).

To optimize this quantitative analysis of malformations of the head skeleton, a variety of distances and angles were evaluated, expanding on a previously reported set evaluated with exposure to 17β-estradiol (Cohen et al. 2014). The different phenotypes induced by the dithiocarbamates and the triazoles supported re-evaluation of the quantification of head skeleton malformation using these two apparently differently acting chemical classes. For that purpose, we compared the quantifiability of the size and direction of the effects and the shape of the dose–response curves for the various parameters between both compound classes. Based on limited variability, intermediate size of effect (enabling detection of even stronger effects), and range of concentrations determining the effect, the M–PQ angle was considered to be most suitable and therefore used in further experiments. This outcome confirmed the M–PQ angle to be an informative parameter as in Cohen et al. (Cohen et al. 2014).

Using the M–PQ angle, a wider set of compounds associated with malformations of the skeleton/cleft palate (Kyriakopoulou et al. 2016; Nielsen et al. 2012) was assessed for effects in zebrafish embryos. Apart from potency variation, this revealed other differences in dose–response fits among compounds. Retinoic acid and dioxin receptor agonists generally produced the highest maximum effect sizes, possibly indicating the specificity of the effect, i.e., the effect could be expressed without inference of other (lethal) effects. This may not be the case when only lower maximal effects are reached (generally without a plateau). Similarly, interference is also suggested by submaximal effect sizes as observed at the highest concentration with various compounds, e.g., maneb and pirimiphos-methyl, which by definition were just sublethal. In cases where the dose–response was determined mainly by the highest concentration (e.g., 2,4 dinitrophenol, boric acid), a small margin can be concluded between induction of specific skeletal effects and general lethal effects. Otherwise, differences between maximal effects may be explained by the specific phenotype, e.g., inhibited outgrowth of the head cartilages compared to dysmorphogenesis (wavy skeleton), which may affect the measured parameter in a different way. Differences in the steepness of the curves, such as the remarkable gentle slope with two of the DTCs and the gentle slope for PCB 126 compared to TCDD, may be due to various interacting toxicokinetic processes, which may amplify or counterbalance each other and the onset of the effect (e.g., through availability at the molecular target) in a different way depending on the chemical structure of the compound. On the other hand, in the re-analysis of historical data, dose–response shapes were found to be homogenous among chemicals in in vitro studies, while a mild among-chemical variation in the steepness parameter seemed to be present in the in vivo studies (Slob and Setzer 2014). Therefore, considering an identical shape of the individual dose–responses, an additional analysis was performed with the combined data set including individual observations of all compounds. Thus, the resulting confidence intervals are smaller than when derived from dose–response analyses per individual compound, and this combined analysis is of particular value to derive relative potency factors, as was exemplified in the dedicated case of cyproconazole and TCDD.

No or very limited effects with compounds that were included on the basis of their occurrence in the CAG skeletal malformation database (Nielsen et al. 2012), such as imidacloprid and 5-fluorouracil, can be due to either sensitivity differences among species or to specific toxicokinetic limitations of the zebrafish embryo model [e.g., limited absorption in the chorion phase; (Kais et al. 2013; Pelka et al. 2017)]. Some experimental confirmation for CAG membership is provided for these two examples, i.e., for imidacloprid in a chick embryo model (Wang et al. 2016) and for 5-fluorouracil in cultured rat embryonic tissue (Shuey et al. 1994). However, these few observations may be too limited as a basis for inclusion in the mammalian CAG skeletal malformations. CAG membership may also be questioned when developmental toxicity is secondory to maternal toxicity (Teixido et al. 2018), which may be the case with the dithiocarbamates, where skeletal variants were induced with maneb in CD1 mice and in Sprague–Dawley rats at a high dose overlapping with maternal toxicity (Beck 1990; Kapp et al. 1991).

Thus, comparative evaluation of a wider set of compounds is informative to assess the specific skeletal toxicity potency of chemicals and, within the aforementioned restrictions, the zebrafish embryo is a helpful model to do so in a quantitative way and with a reasonable throughput. However, in the context of hazard identification for human risk assessment, one-to-one translation should be avoided, and the zebrafish embryo would be supportive (with the specific advantage of a whole organism) in a battery of test models. A recommended next step is to implement this particular application of the zebrafish embryo in an adverse outcome pathway (AOP) or a network of AOPs, as a test to evaluate specific key events, together with other *in silico* and in vitro testing along the same pathway (Villeneuve et al. 2014). Such combined testing can reduce uncertainty regarding the apical adverse outcome and increase human relevance.

For application of the M–PQ angle in mixture analysis, a case was made by combining two compounds which induced a reasonable size of the effect at intermediate concentrations in the dose–response curve, i.e., cyproconazole and TCDD. These compounds have a dissimilar mode of action, which makes the question to possible concentration addition more relevant than in cases of similar mode of action (Nielsen et al. 2012). In the combined analysis with single compounds, the mixtures did not deviate systematically from the overall dose–response curve, supporting that in this case, the combined effect could be safely predicted by the concentration addition model. The present design with a limited number of data points of the various mixture conditions (equipotency or relative excess of one of the compounds) should be sufficient to suggest dominance of either compound, which could be caused by direct interaction between compounds or interaction along the toxicity pathway in the organism (Cassee et al. 1998). Such suggested dominance should be further confirmed in a repeat experiment focusing on that concerning condition.

We have shown that measurement of the M–PQ angle can be used to assess the effects of single compounds and a binary mixture on malformations of the head skeleton in zebrafish embryos. Based on the current data, we conclude that our method is suitable to assess the effects of compounds representing a good variety of chemical classes included in the CAG skeletal malformations and associated databases, and in addition to confirm concentration addition in a test case of a binary mixture. More data on mixtures may reveal the potential of the method to analyze combination effects of compounds of varying classes and/or varying modes of action, to further test the hypothesis that concentration addition safely predicts such combination effects.

In conclusion, developmental skeletal malformations can be quantitatively analyzed using alcian blue staining in 120 hpf zebrafish embryos, by measuring the Meckel’s–palatoquadrate angle. Visual assessment as an additional check in cases of absence of M–PQ angle effects did not reveal missed cartilage malformations. Most compounds representing a wide variety of chemical classes included in the EFSA CAG skeletal malformations induced effects in zebrafish embryos (ZFE), and potential explanations for the absence of effects with some compounds could be generated. The method may thus contribute to more precise definition of CAG membership of compounds, and the most optimal way to proceed is to include the ZFE in a suite of assays, preferably in a structured way such as the AOP approach. Finally, the method appeared suitable to analyze the effect of a combination of compounds in a binary mixture, and in the examined test case confirmed that the two compounds from different chemical classes induced the effect through the concentration addition model.
